# Cardiovascular magnetic resonance compatible physical model of the left ventricle for multi-modality characterization of wall motion and hemodynamics

**DOI:** 10.1186/s12968-015-0154-9

**Published:** 2015-06-26

**Authors:** Ikechukwu U. Okafor, Arvind Santhanakrishnan, Brandon D. Chaffins, Lucia Mirabella, John N. Oshinski, Ajit P. Yoganathan

**Affiliations:** School of Chemical & Biomolecular Engineering, Georgia Institute of Technology, Atlanta, GA USA; School of Mechanical & Aerospace Engineering, Oklahoma State University, Stillwater, OK USA; Wallace H. Coulter Department of Biomedical Engineering, Georgia Institute of Technology and Emory University, Atlanta, GA USA; Department of Radiology and Imaging Sciences, School of Medicine, Emory University, Atlanta, GA USA

**Keywords:** Cardiovascular magnetic resonance, Left ventricle phantom, MR segmentation, MR reconstruction, MR validation, Particle image velocimetry, Stereo-photogrammetry, FSI validation

## Abstract

**Background:**

The development of clinically applicable fluid-structure interaction (FSI) models of the left heart is inherently challenging when using *in vivo* cardiovascular magnetic resonance (CMR) data for validation, due to the lack of a well-controlled system where detailed measurements of the ventricular wall motion and flow field are available *a priori*. The purpose of this study was to (a) develop a clinically relevant, CMR-compatible left heart physical model; and (b) compare the left ventricular (LV) volume reconstructions and hemodynamic data obtained using CMR to laboratory-based experimental modalities.

**Methods:**

The LV was constructed from optically clear flexible silicone rubber. The geometry was based off a healthy patient’s LV geometry during peak systole. The LV phantom was attached to a left heart simulator consisting of an aorta, atrium, and systemic resistance and compliance elements. Experiments were conducted for heart rate of 70 bpm. Wall motion measurements were obtained using high speed stereo-photogrammetry (SP) and cine-CMR, while flow field measurements were obtained using digital particle image velocimetry (DPIV) and phase-contrast magnetic resonance (PC-CMR).

**Results:**

The model reproduced physiologically accurate hemodynamics (aortic pressure = 120/80 mmHg; cardiac output = 3.5 L/min). DPIV and PC-CMR results of the center plane flow within the ventricle matched, both qualitatively and quantitatively, with flow from the atrium into the LV having a velocity of about 1.15 m/s for both modalities. The normalized LV volume through the cardiac cycle computed from CMR data matched closely to that from SP. The mean difference between CMR and SP was 5.5 ± 3.7 %.

**Conclusions:**

The model presented here can thus be used for the purposes of: (a) acquiring CMR data for validation of FSI simulations, (b) determining accuracy of cine-CMR reconstruction methods, and (c) conducting investigations of the effects of altering anatomical variables on LV function under normal and disease conditions.

## Background

Heart failure is a significant problem in the western world and is present in over 2 % of the adult population over the age of 65 [1]. Left Ventricle (LV) structural abnormalities (e.g., dilated cardiomyopathy [2]), valvular pathologies (e.g., aortic stenosis), abnormalities in electrical conduction (e.g., LV dyssynchrony), and hypertension can act as some of the causative agents of heart failure. Challenges with timely diagnosis of heart failure exist when using traditional clinical metrics, as is the case in diastolic heart failure with preserved ejection fraction [3]. Detailed studies examining the complex mechanical interactions between the various anatomical structures (left atrium, LV, aorta, and corresponding valves) on the pumping function are thus needed at the isolated structural levels and collective organ level. Such investigations can aid in improving clinical outcomes by identifying more accurate diagnostic measures for earlier intervention, as well as in optimizing treatment options *a priori*.

From a physiological standpoint, several recent studies using clinical data [2, 4–11], *in vitro* models [12–18], and computational simulations [16, 19–27] have pointed to the importance of examining intra-ventricular fluid dynamics for potential use as a diagnostic metric of cardiac health. Intricately coupled to the intra-ventricular fluid flow are the wall motion of the LV and valvular kinematics, which can be affected across multiple pathological conditions observed in heart failure patients [2, 9]. From a clinical perspective, the use of non-invasive medical imaging techniques (including cardiovascular magnetic resonance (CMR) [4, 6, 8, 11] and echocardiography [2, 7, 9, 10]) monitor and detect of abnormalities in ventricular wall motion, valve operation, and intra-ventricular flow patterns. The data obtained from these non-invasive techniques can also be used in computational models [21, 23, 24, 28] in order to provide patient specific treatment options.

The main obstacles to obtaining high-resolution *in vivo* medical imaging data from volunteers and patients are high operational costs and the length of the scan time [29]. It must be noted that the accuracy of cine CMR reconstruction (which is affected by the spatial and temporal resolution of the acquisition) is of foremost importance when provided as input data for FSI methods. As a result, the techniques used in the reconstruction of cine CMR and phase-contrast CMR (PC-CMR) data for LV wall motion and flow fields, respectively, need to be validated. In this regard, the use of *in vitro* platforms can provide a more straightforward, controllable means of obtaining high-resolution experimental data to use in the testing, development, and validation of imaging-based cardiac FSI and CFD models [30, 31].

The research efforts presented in this paper specifically address the need for versatile *in vitro* experimental model of the LV that can be used to obtain data across multiple modalities available across bench-top and clinical practice, so as to compare the relative accuracies of the modalities and for eventual use in providing data for validation of FSI models of the left heart. The specific goals of the study presented herein were to: (a) develop an *in vitro* left heart simulator using a flexible-walled LV physical model that is CMR compatible, and (b) compare the LV volume reconstructions from cine steady-state free precession (SSFP) to laboratory values obtained from high-speed stereo-photogrammetry (SP) and (c) compare the LV hemodynamics from PC-CMR to laboratory values obtained from particle image velocimetry (DPIV) and flow probes (FP). These sets of information will allow for the downstream application of the *in vitro* model in providing CMR data for validation of FSI simulations as well as in comparing the relative accuracies of reconstruction methods used to process anatomical CMR images.

## Methods

### LV physical model

#### Design and construction

**Fig. 1:**
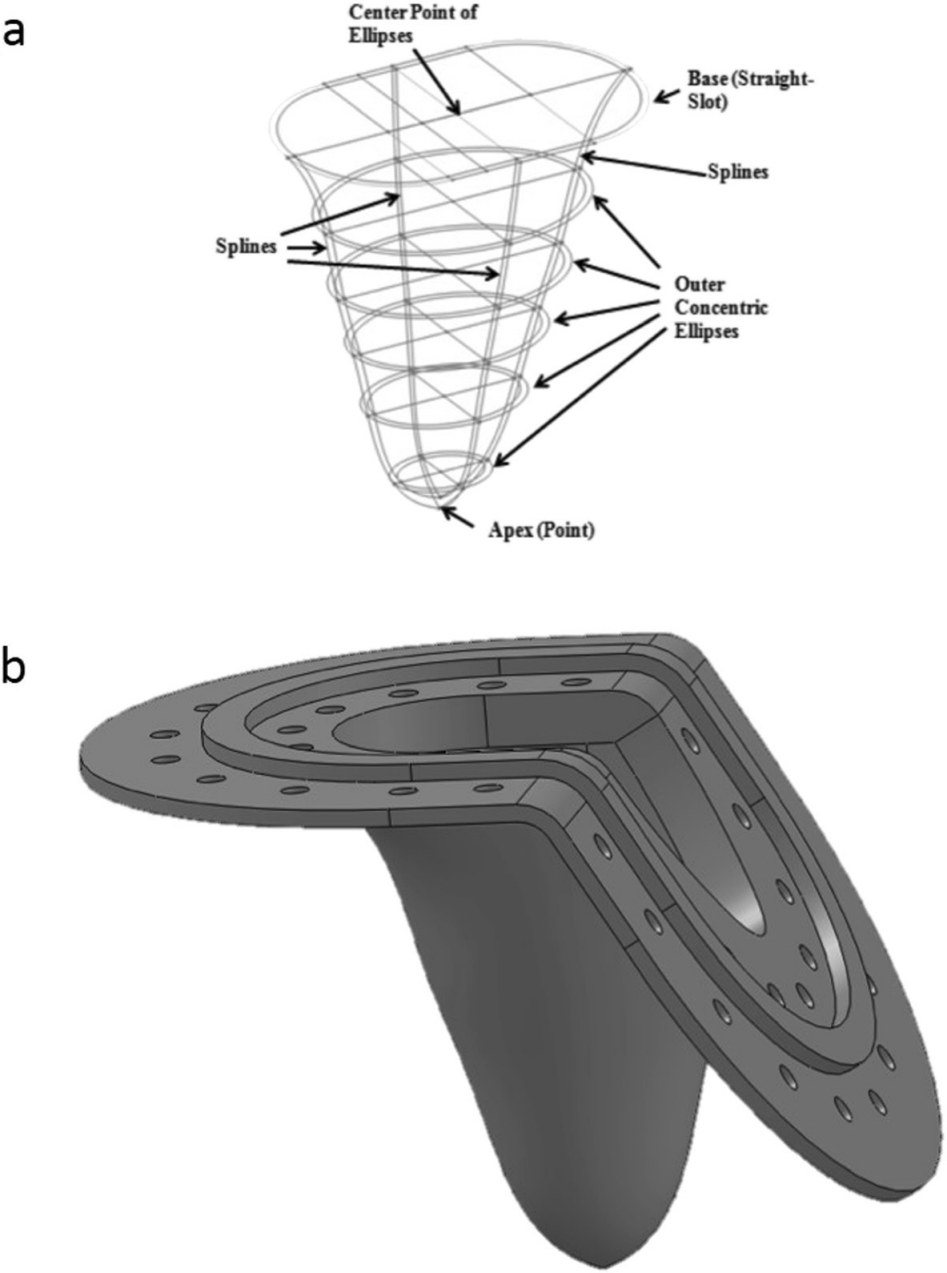
Anatomical physical model of the flexible-walled LV: design of the model using a series of concentric ellipses connected via splines is shown in (**a**), and (**b**) shows the 3D schematic of the geometry.

The left ventricular geometry was generated in Solidworks^TM^ (Dassault Systèmes Solidworks Corporation, Waltham, MA, USA) by constructing a series of concentric ellipses that were fit to LV endocardial borders traced on 5 cine steady-state free procession (SSFP), short axis slices at peak systolic phase of the cardiac cycle acquired in a healthy subject’s (Fig. 1). A 125° cut was made at the base of the ventricle such that the mitral and aortic annular planes matched *in vivo* conditions [32]. The design was sent to a third party company (VenAir, Terrassa, Spain) for tool building and casting. Silicone, with a shore hardness of 42A and a thickness of 0.159 cm, was used as the material for the ventricle casting. This hardness was chosen to provide the flexibility and durability needed for pumping function, while simultaneously to allow optical access for flow visualization inside the ventricle. The patient data collected for the construction of the LV was approved by the institutional review board (IRB# H09236).

#### Flow loop setup

**Fig. 2:**
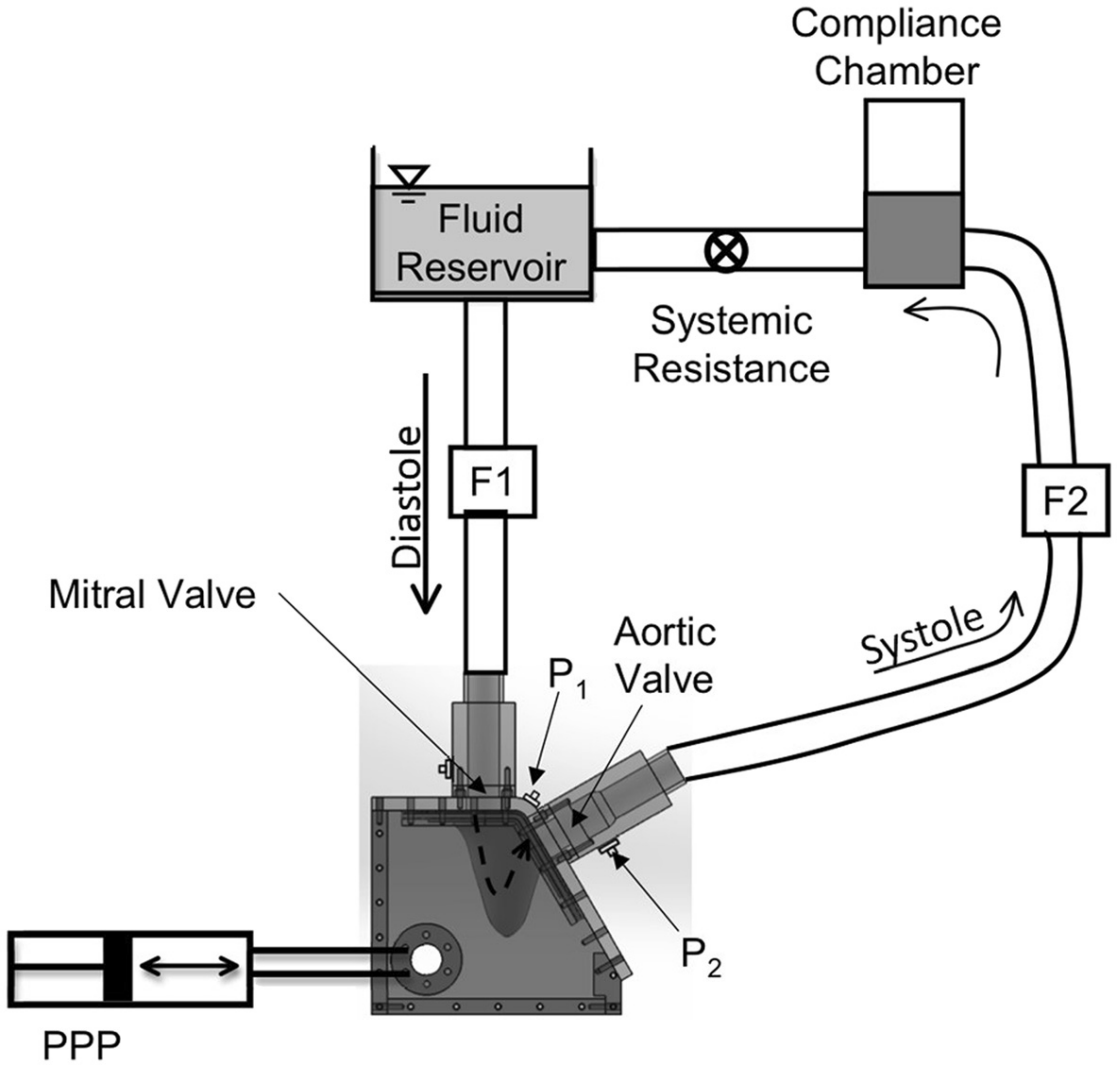
Schematic of the *in vitro* LV flow circuit. The LV physical model is enclosed within an acrylic box filled with water-glycerin solution. Expansion and contraction of the flexible-walled LV model is accomplished via periodic pressure fluctuations of the enclosing fluid using a programmable piston pump (PPP). Flow probes F1 and F2 are used to measure mitral and aortic flow rates, respectively. Measurement locations of two transducers for measurement of LV (P1) and aortic pressures (P2) are indicated. St. Jude Regent BMHVs were used in the mitral and aortic valve positions. The flow direction through the LV model is indicated using a dashed arrow.

The LV model was placed inside an acrylic housing that was filled with a 36 % by volume glycerin solution in water (the same fluid as inside the LV model). This solution was used as a blood analogue fluid that mimics the viscosity of blood at 37 °C and closely matches the refractive index of acrylic. Two 23 mm St. Jude Medical Regent^TM^ bileaflet mechanical heart valves (BMHVs) were placed in the model, one at the mitral annulus and one at aortic annulus to ensure unidirectional fluid flow. Figure 2 illustrates the flow system. A programmable piston pump (PPP; Vivitro Systems Inc., Victoria, Canada) was used to induce the LV wall motion by altering the pressure of the fluid in the space between the interior of acrylic chamber housing the LV model and the exterior of the LV model. The LV wall motion in turn generates pulsatile fluid flow into and out of the ventricle through the valves. Absolute pressures were measured at the atrial, ventricular, and aortic positions using pressure transducers (Utah Medical Products Inc., Midvale, UT). Volumetric flow rates into and out of the ventricle were measured using ultrasonic flow probes (Transonic Systems Inc., Ithaca, NY). However, it should be noted that during the CMR experiments, the flow probes were replaced with rigid pipes of the same length and internal diameter of the probes. The ventricular wall motion was studied under physiologic hemodynamic conditions (120/80 mmHg systemic pressure, 3.5 L/min average cardiac output at a heart rate of 70 beats/min).

### Stereo photogrammetry

#### Experimental setup of high-speed cameras

**Fig. 3:**
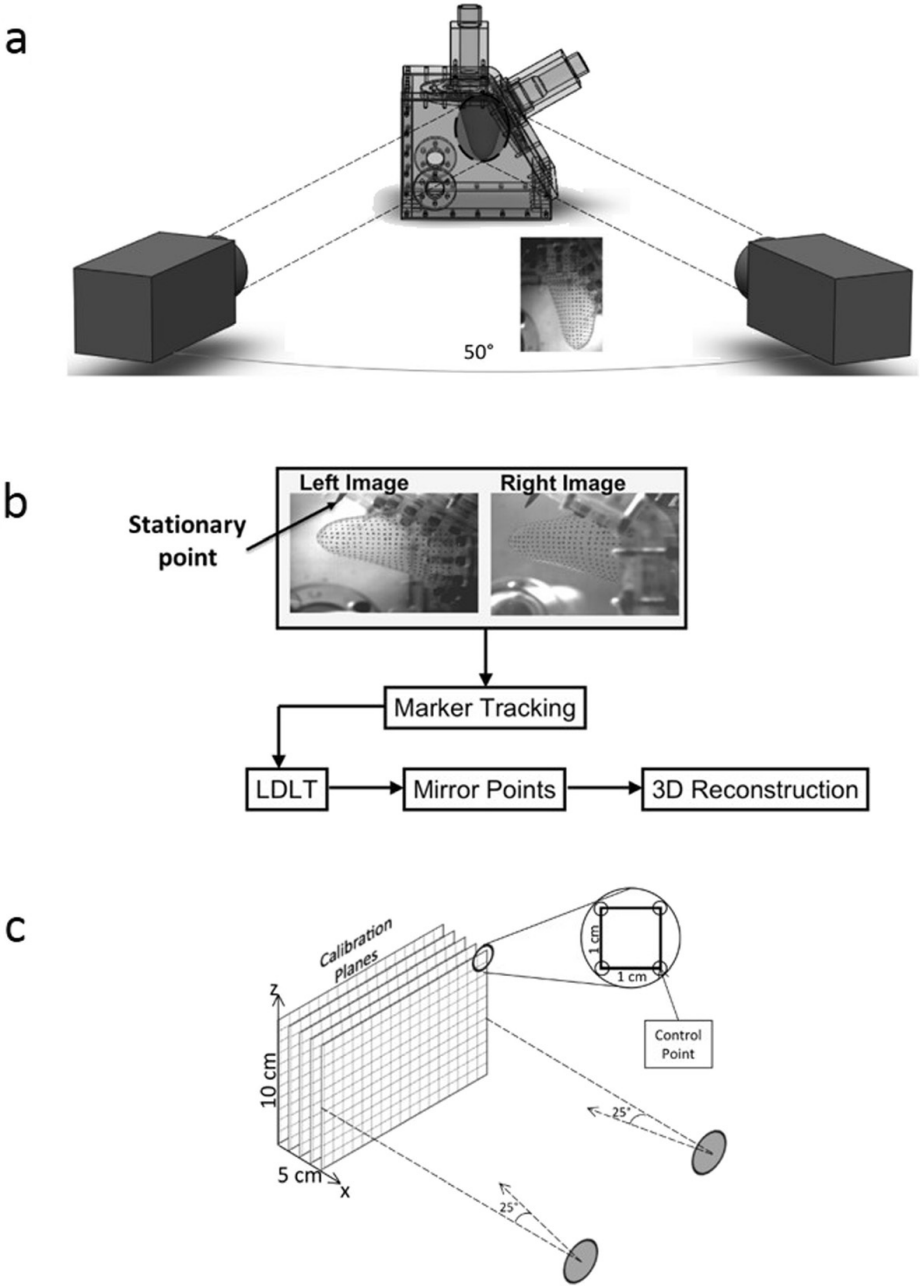
Experimental setup for conducting stereo-photogrammetry and post-processing: (**a**) shows the arrangement of the dual high-speed cameras relative to the LV chamber, (**b**) shows workflow used to process the raw image data and obtain the volumetric reconstruction of the 3D geometry, and (**c**) shows the calibration target used and its position relative to the cameras.

To assess wall motion in the laboratory, dual camera stereo-photogrammetry was performed using two high-speed monochromatic cameras (Model A504K, Basler Vision Technologies, Exton, PA; 1280 × 1024 pixels) with Nikon macro lenses (60 mm, f2.8; Nikon, Melville, NY). A grid of circular markers, with a 4 mm by 4 mm discretization, was printed on one side of the outer surface of the ventricle. Each of the markers was approximately 2 mm in diameter, Fig. 3(a). During experiments, the cameras were externally triggered at the same time as the pulse duplicator system to synchronize the camera images with the hemodynamic (pressures and flow rates) acquisition. 214 time points were acquired during a cardiac cycle for a total of 15 cycles. The 15 cycles of data for each tracked marker point were ensemble averaged to give one cycle.

#### Calibration

Prior to each experiment, a 3D spatial calibration was performed using the localized direct linear transformation method (LDLT) [33]. For the calibration, the acrylic chamber that housed the LV was filled with the same 36 % by volume water-glycerin solution. As shown in Fig. 3(c), a sheet with a 1 cm grid was placed inside the chamber and traversed in the x-direction until the volume occupied by the ventricle was covered. The cameras were positioned such that all control points in the volume occupied by the ventricle could be captured.

#### Reconstruction of volume

A pin (see Fig. 3(b) served as a stationary point in the field of view of both cameras, around which the reconstructed points were mirrored. This allowed for the generation of the LV volume under the assumption that the LV contraction and relaxation was symmetric along the long axis of the left-ventricular outflow tract center plane. A surface was then fit to the points at each time point using Geomagic 3D Software (Geosystems, Rockhill SC). The surfaces were then imported into Paraview (Kitware, Clifton Park NY) where the volumes were extracted.

### Digital Particle Image Velocimetry (DPIV)

#### Experimental setup

**Fig. 4:**
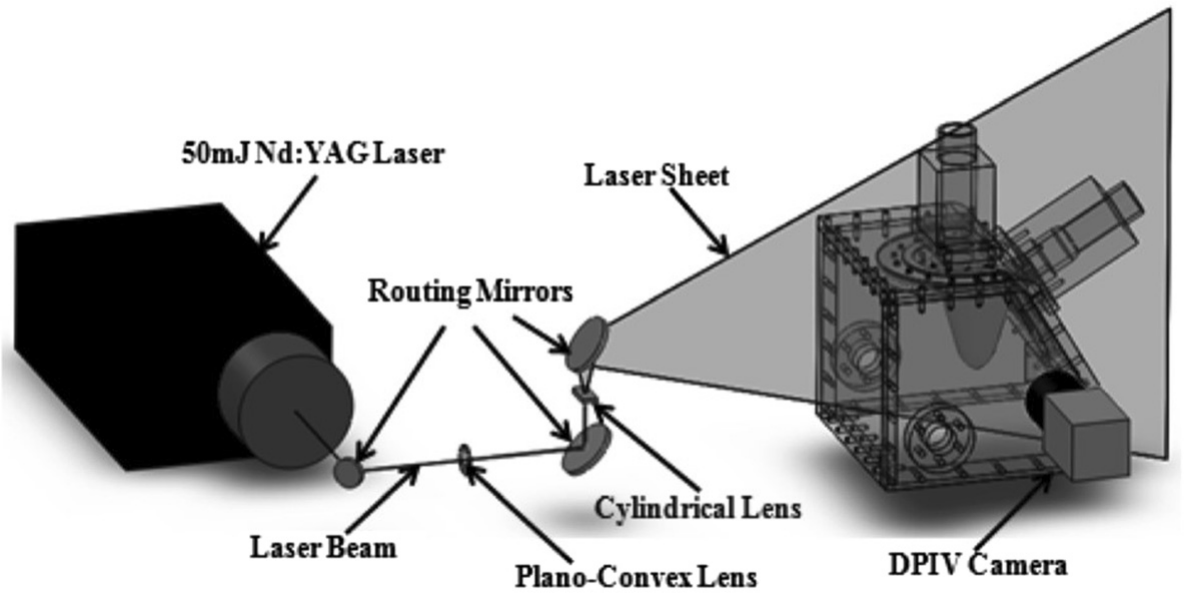
Experimental setup of camera, laser and optics used for conducting DPIV measurements on the LV physical model. The laser beam was routed to a plano-convex lens for focusing purpose, followed by a cylindrical lens for generating a light sheet. An arrangement of three mirrors was used for routing the laser beam through the lenses and reflecting the light sheet onto the LV physical model.

To assess hemodynamics in the laboratory, DPIV was used to quantitatively visualize the flow patterns through a long axis plane of the ventricle corresponding to a 2-chamber long-axis view. Figure 4 shows the schematic of the DPIV set up. The BMHV used in the mitral position was located upstream of the mitral annulus, compared to the placement at the level of the mitral annulus in experiments using other experimental modalities. This change in mitral valve location for DPIV experiments was done to examine the flow through the mitral orifice without including the leaflets. This allowed us to compare to *in vivo* flow fields from previous studies without being affected by the flow through the “three-jet” orifice characteristic of the SJM Regent BMHV design. This change in mitral valve placement was the only difference in the setup of the LV model between DPIV and all other experimental modalities used in this study. The area of the valveless mitral annulus was 3.0 cm^2^. The fluid inside the ventricle was seeded with neutrally buoyant fluorescent particles (PMMA with RhB dye, 1–20 lm, Dantec Dynamics; Denmark) and was illuminated using a dual pulsed, 1 mm thick, laser light sheet (Nd:YAG lasers, 17 mJ/pulse, 532 nm, ESI Inc.; Portland, OR). The particles were imaged using a Nikon Micro-Nikkor 60 mm lens attached to a CCD camera (Imager Pro X 2 M, LaVision, Germany, Imager Pro, 1600 × 1200 pixels).

Phase-locked DPIV images were acquired for 34 time points in the cardiac cycle, each spaced by 25 ms. The mean flow field for each phase point in the cardiac cycle was computed via ensemble averaging of 200 instantaneous DPIV image pairs acquired from 200 cardiac cycles. The time spacing between image pairs (*dt*) was in the range of 800 – 1200 μs across all the phase points in the cardiac cycle, where the phase-specific *dt* value was selected to allow 5 – 8 pixels of particle displacement.

#### Processing

The images were preprocessed in DaVis 7.2 (LaVision, Germany) by performing a sliding background subtraction of a scale length of 5 pixels. Particle cross correlation was performed on the images using dual-pass interrogation with decreasing window size (64 × 64 to 32 × 32 pixels; 50 % overlap). Vectors were deleted if peak ratio, Q, was less than 1.2; interpolation was performed to fill up all empty spaces.

### CMR

#### Cine-SSFP for LV motion

**Fig. 5:**
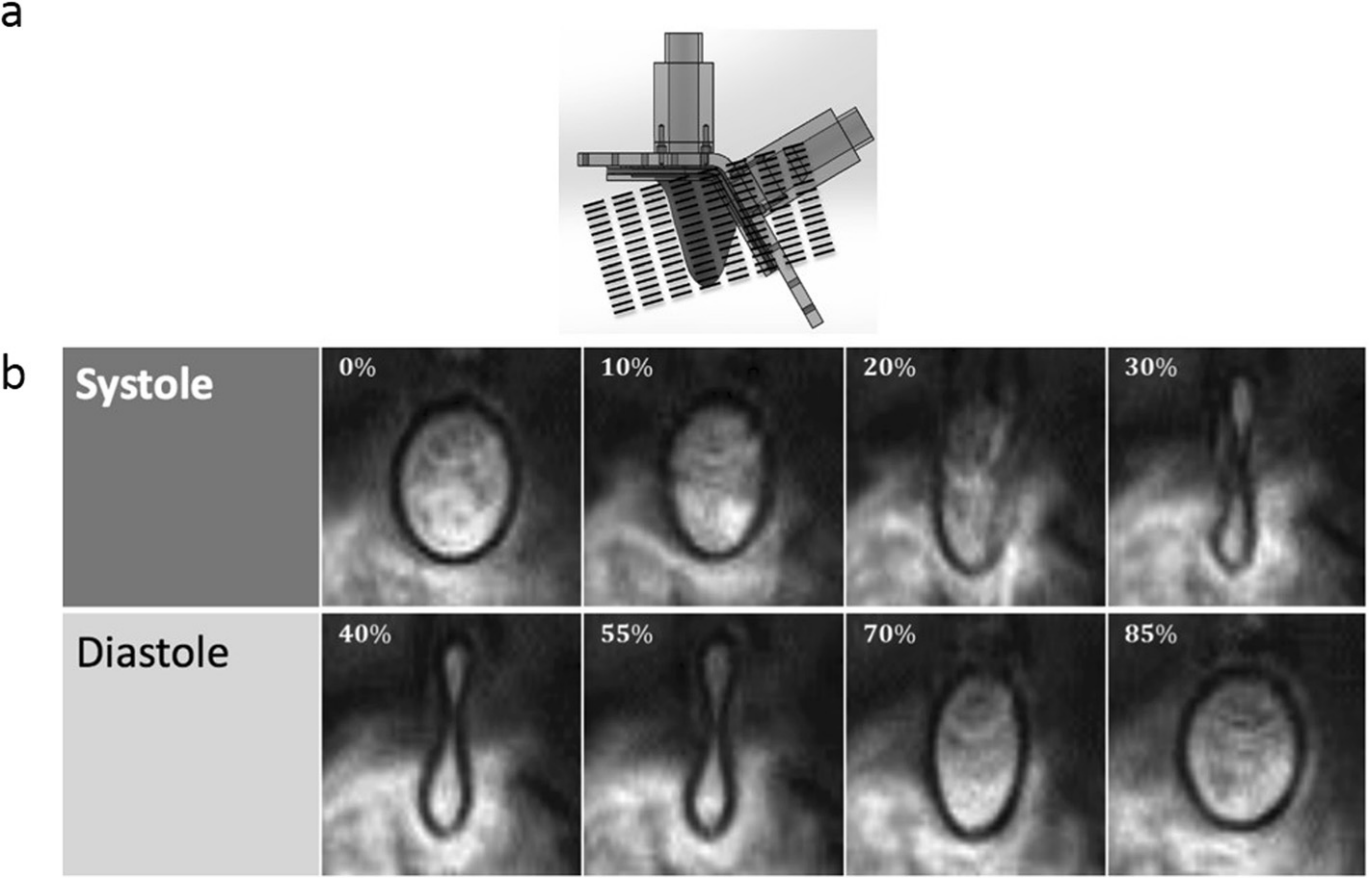
Cine-CMR measurements of the LV physical model wall motion: (**a**) shows the orientation of the 15 planes (or short axis slices) used for acquiring anatomical cine-CMR images for quantifying wall motion, and (**b**) shows representative images across both systolic and diastolic phases of the cardiac cycle (time point is indicated as percentage of cardiac cycle period). For (**b**), 128 phases were acquired across the cardiac cycle, with 0 mm spacing between slices.

The heart model was examined on a 3 T Siemens scanner to evaluate LV wall motion. Contiguous short axis images slices were acquired using a cine balanced steady-state free precession (SSFP) sequence. A six-element phase array body coil along with elements from the spine coil built into the table. The acquisition sequence was retrospectively ECG-gated using an external TTL pulse sent to an ECG-pulse conversion box which triggered the CMR scanner through the ECG gating module. An acceleration factor of 2 was used using the GRAPPA technique. The SSFP cine images were acquired with an in-plane resolution of 1.2 by 1.2 mm, a slice thickness of 6 mm, and a reconstructed temporal resolution of 7 ms (128 frames/cycle). Two signal averages were acquired resulting in an acquisition time of 3:28 seconds per slice for the PC-CMR sequence. TE (echo time) = 3.3 milliseconds. Two segments were acquired per cardiac phase per heartbeat, yielding an effect TR (temporal resolution) of 24 milliseconds. To cover the entire LV, 15 slices were acquired with no gap. Figure 5a shows the orientation at which the slices were acquired.

#### Image segmentation

Using a region based active contour model developed by Wang *et al.* [34], the LV geometry at every phase was segmented from the short axis cine MR images. Point clouds were extracted from the binary images generated from segmentation and surface fit to them and smoothed using the ‘relax’ feature on Geomagic 3D Software (Geosystems, Rockhill SC). The internal volumes of the LV were calculated in MATLAB (MathWorks, Natick MA) by multiplying the areas of the binary images with the corresponding slice thickness.

#### Phase contrast magnetic resonance (PC-CMR)

**Fig. 6:**
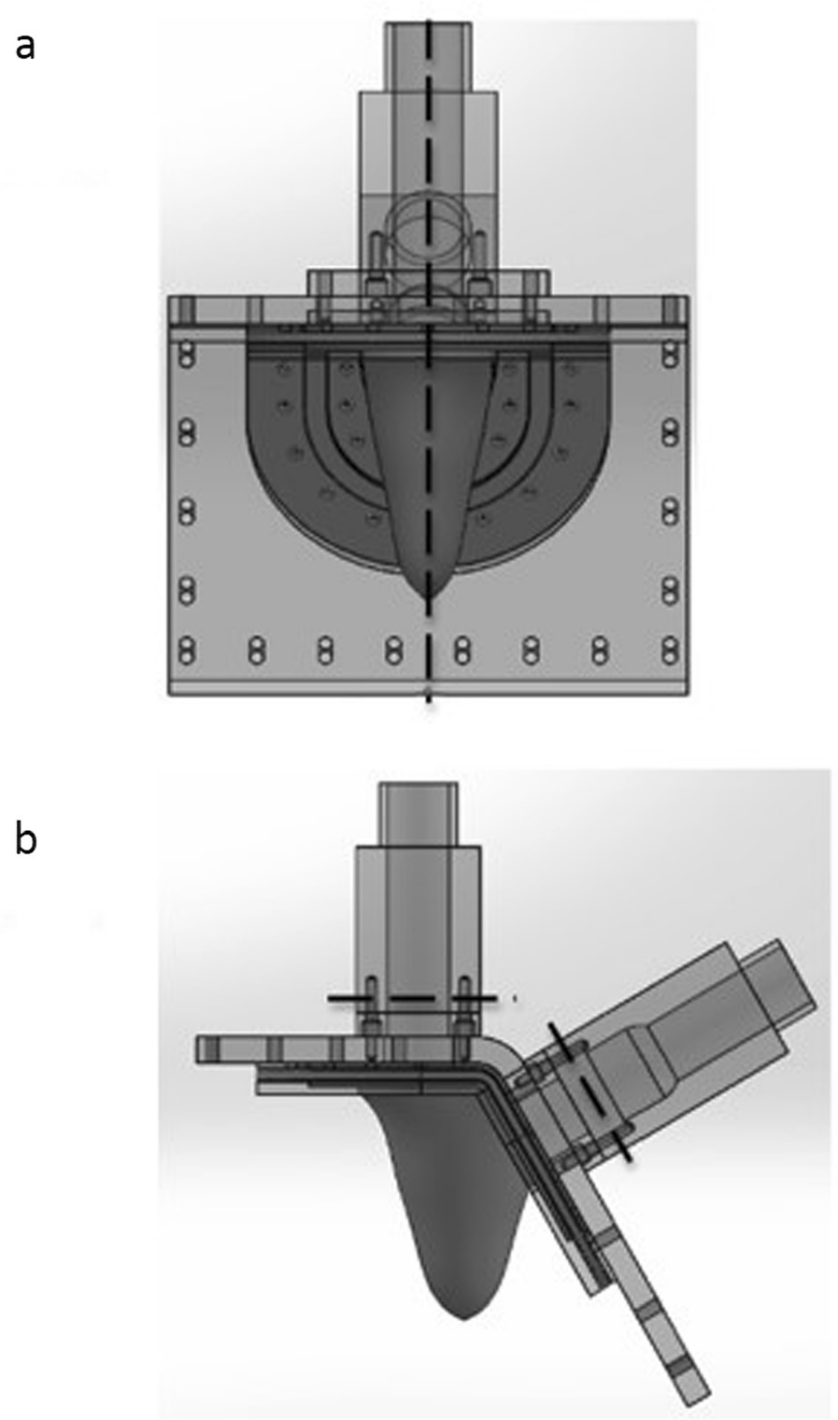
Locations of the planes (relative to the LV physical model) used for acquiring PC-CMR measurements: (**a**) shows the plane used for acquiring in plane velocity measurements of the flow through the LV model, and (**b**) shows the locations of the two planes used for acquiring PC-CMR measurements of normal component of velocity (through-plane) upstream of the mitral and aortic valves.

Mitral and aortic valve flows were acquired using 2D PC-CMR sequence, encoding a single velocity direction (through plane). An acquisition using in plane velocity (2 directions) was performed in order to extract the 2D velocity field of the left ventricular outflow tract (LVOT) plane. The imaging planes that were acquired is shown in Fig. 6. The PC-CMR sequence used was a retrospectively ECG-gated gradient echo sequence with a slice thickness of 6 mm and the velocity encoding was 150 cm/s. 20 phases were acquired through the cardiac cycle at each position. A spline interpolation was performed using the inbuilt MATLAB (MathWorks, Natick MA) function in order to match the discretization of the PC-CMR data with that of the flow probes.

### Volume reconstruction comparison

PC-CMR and flow probes are similar modalities in that they provide flow (volume/time) information across the mitral and aortic valves as a function of time. The volume within the LV at each time instant was thus calculated using Equation 1 below:

1 $$ V(t) = {V}_{change}+ constant = {\displaystyle \underset{0}{\overset{t}{\int }}}{\left.\frac{dV}{dt}\right|}_{LV}dt=\kern0.5em {\displaystyle \underset{0}{\overset{t}{\int }}}\left[{\left.\frac{dV}{dt}\right|}_{AV} - {\left.\frac{dV}{dt}\right|}_{MV}\right]dt $$

where LV, AV, MV, and t are the left ventricle, aortic valve, mitral valve, and time of cardiac cycle, respectively. The value of ‘constant’ is the end systolic volume of the LV, which was obtained from either the CMR or SP experiments.

For the SP and CMR modalities, the reconstructions directly provide the instantaneous volumes at each time instant. The volumes were normalized by their respective local maxima such that a one-to-one comparison between modalities could be conducted. In this study, normalized volume was denoted as $\overline{V(t)}$. The normalized volumes through the LV from SP and CMR were compared to equivalent values obtained using flow probes and PC-CMR, via calculation of the absolute value of the relative difference in $\overline{V(t)}$ between modalities.

## Results

### Ventricular wall motion

Figure 5 shows the CMR images obtained at multiple time points during the cardiac cycle. At some time points (total of 7) during the early diastolic and peak systolic period of the cardiac cycle, there was blurring of the LV wall in the images; hence, segmentation was not possible. The rest of the phases (~95 %) were reconstructed to produce 3D volumes of the ventricle through time. The maximum and minimum volumes of the LV through this method were found to be 65 and 31 mL respectively, giving an ejection fraction of 52.3 %.

Due to the high temporal frequency of stereo-photogrammetry, none of the phases acquired experienced the same blurring issue as was found with CMR. Only half of the ventricle wall was imaged and centerline symmetry was assumed such that the marker points were mirrored across a stationary pin located at the line of symmetry to generate the 3D volume. The maximum and minimum volumes of the LV through the SP method were found to be 62 and 33 mL respectively, giving an ejection fraction of 46.8 %.

### Intra-ventricular flow field

**Fig. 7:**
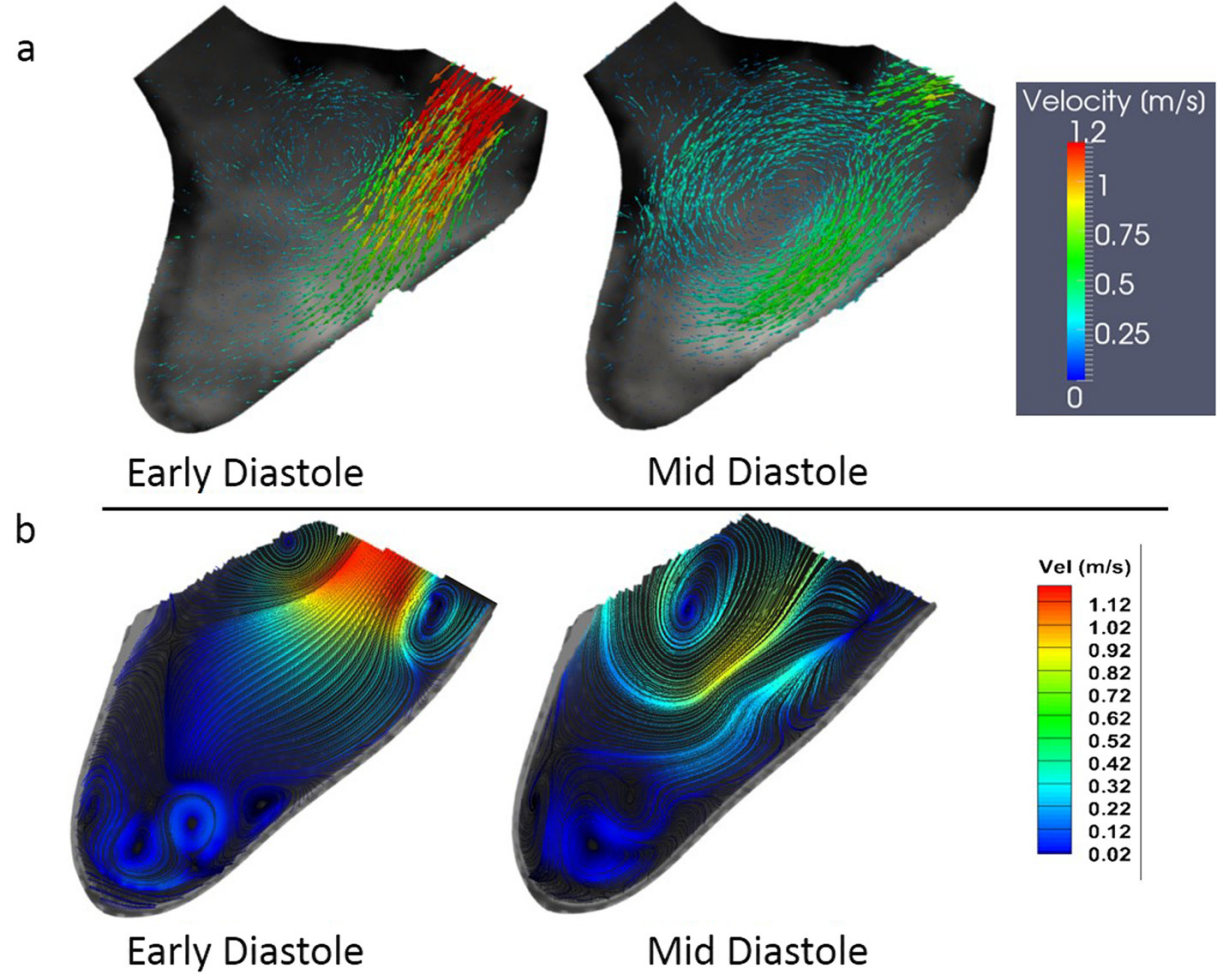
DPIV and PC-CMR measurements on the LV physical model during the early and mid-diastolic phases of the cardiac cycle: (**a**) PC-CMR velocity vectors (**b**) DPIV streamlines colored with velocity magnitudes.

DPIV was performed on the central long-axis plane of the LV. Figure 7 shows the velocity field from PC-CMR (a) and DPIV (b) of two representative time points during diastole (early and mid-diastole in the cardiac cycle). In both DPIV and PC-CMR velocity fields there was a single central jet is shown from the atrium into the LV with a velocity of about 1.15 m/s. In the DPIV velocity field reconstruction, two counter rotating vortices are observed in the flow field, advecting ahead of the trailing jet. The far field velocity magnitudes are comparatively lower due to lack of mixing with the apical flow, which was expected as the time point is in early vortex ring propagation stage [10, 20]. In PC-CMR velocity field reconstruction, the magnitudes of velocities observed are similar to DPIV both in the early and mid-diastolic phase of the cardiac cycle (Fig. 7). The overall flow structure observed was similar between two modalities; however, the two counter rotating vortices formed during diastole were not well resolved in PC-CMR. This could be due to the relative coarser resolution of PC-CMR. The resolution of DPIV was much greater, hence, it is able to resolve much finer flow structures.

### Hemodynamics

**Fig. 8:**
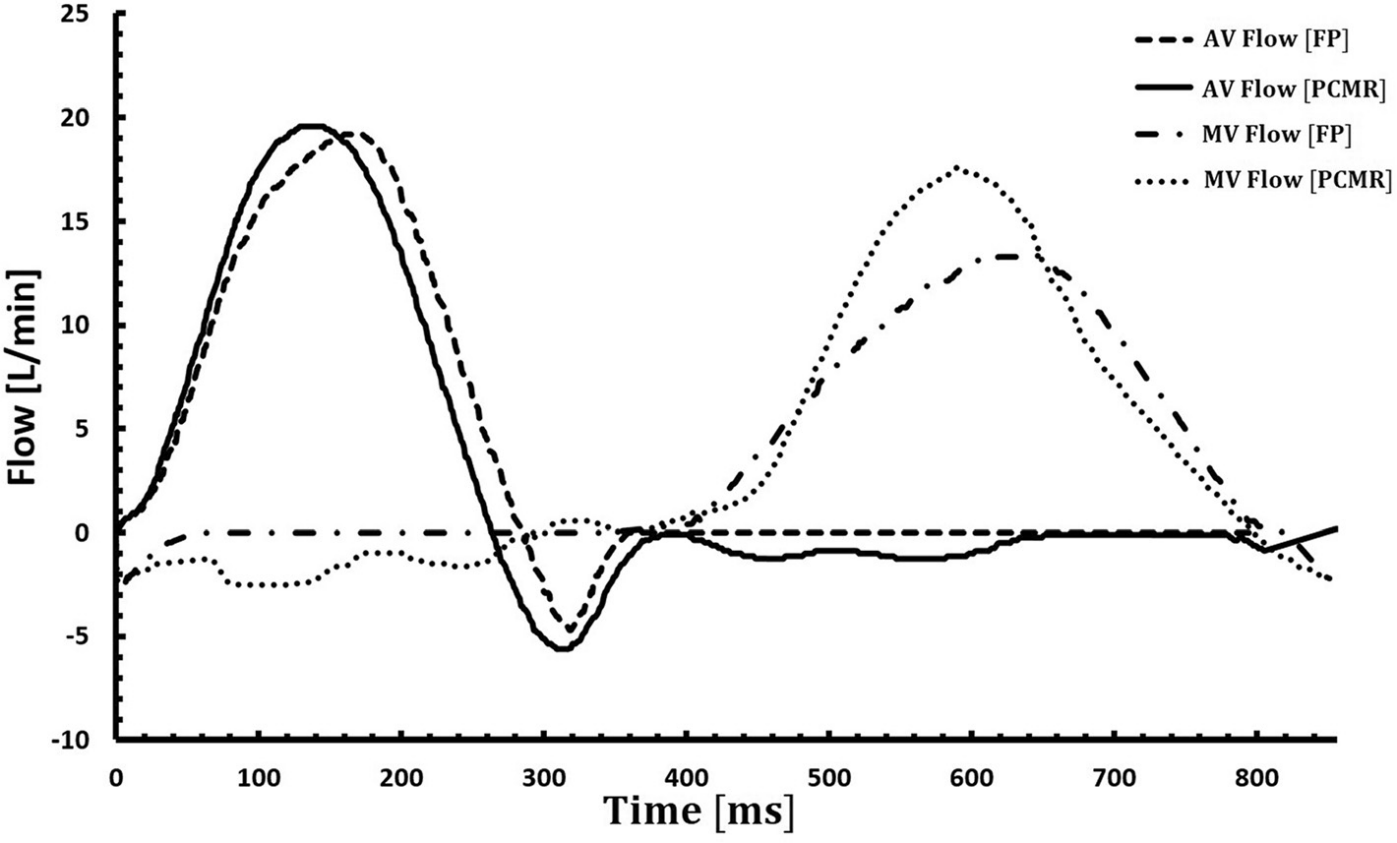
Mitral and aortic flow rates obtained via FP during the stereo-photogrammetry (SP) as well as the flow curves from PC-CMR acquisition on the LV physical model.

Figure 8 compares the flow curves obtained during stereo-photogrammetry experiments via flow probes to those obtained during PC-CMR experiments. The flow curves obtained from the flow probes were averaged over 15 cardiac cycles. From the PC-CMR experiments, at the aortic plane, the peak aortic flow rate obtained was 20 L/min and the peak mitral flow rate was 17.5 L/min. The overall magnitudes of the flow rates between the FP and PC-CMR were similar, with the cardiac outputs for both modalities averaging at 3.5 L/min. The PC-CMR flow curves showed slight mitral and aortic regurgitation.

### Volume comparison: CMR and stereo-photogrammetry

**Fig. 9:**
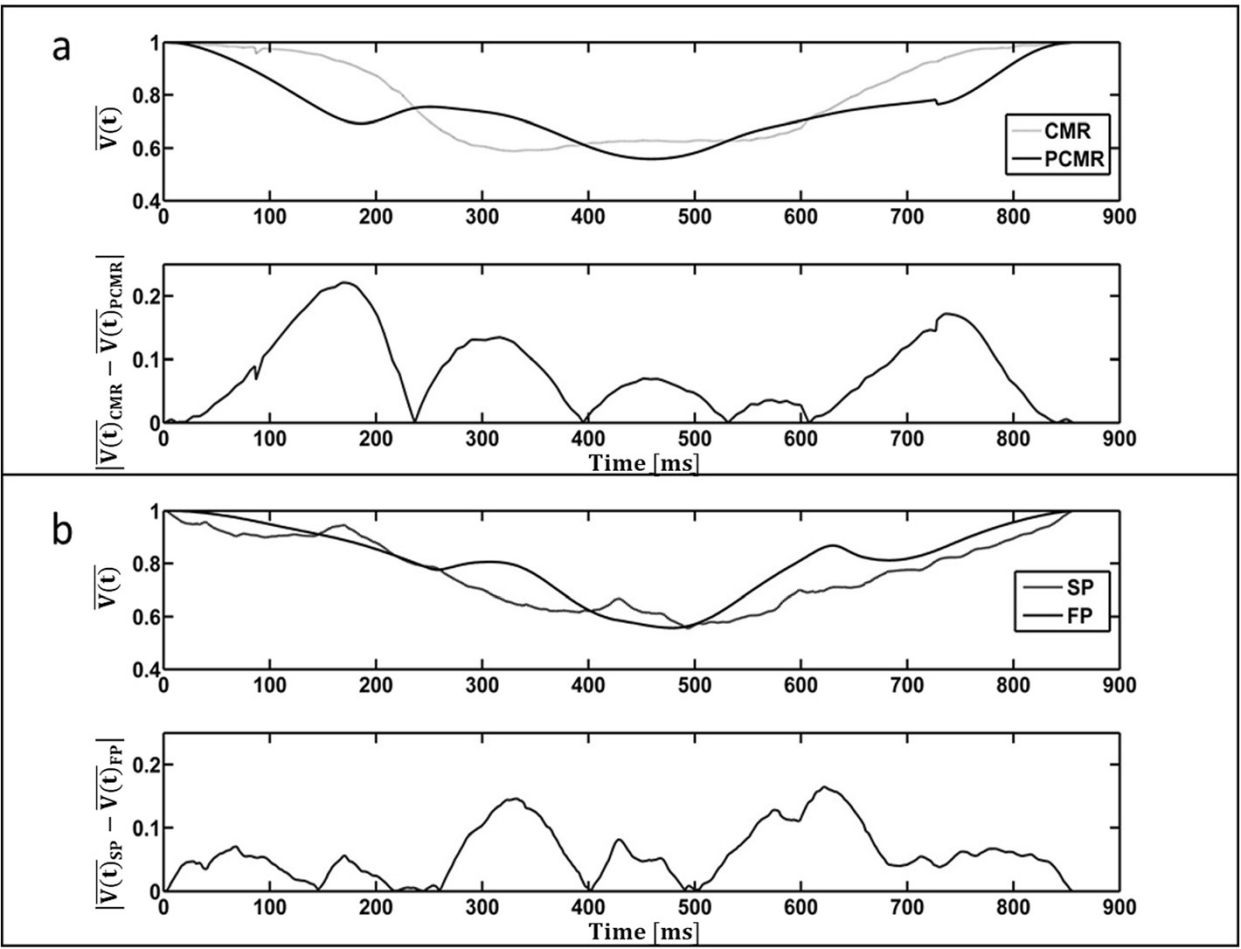
Comparison of the normalized volume $\overline{\left(V(t)\right)}$ through the LV, as a function of the cardiac cycle time, between (**a**) cardiac magnetic resonance $\overline{\left(V\right.(t)}\left.{}_{CMR}\right)$ and phase contrast cardiac magnetic resonance $\overline{\left(V\right.(t)}\left.{}_{PCMR}\right),$ and (**b**) stereo-photogrammetry $\overline{\left(V\right.(t)}\left.{}_{SP}\right)$ and inline flow probes $\overline{\left(V\right.(t)}\left.{}_{FP}\right)$. The absolute values of the difference between the modalities compared in (**a**) and (**b**) are also shown as a function of the cardiac cycle.

**Fig. 10:**
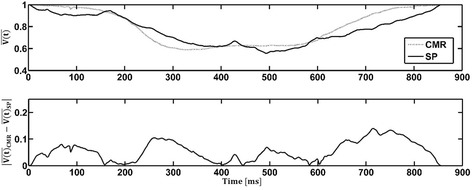
Top panel: comparison of the normalized volume within the LV physical model, as a function of the cardiac cycle, obtained using volume reconstruction from stereo-photogrammetry $\overline{\left(V\right.(t)}\left.{}_{SP}\right)$ and cine-CMR $\overline{\left(V\right.(t)}\left.{}_{CMR}\right)$ modalities. Bottom panel: the absolute value of the difference between ${\overline{V(t)}}_{CMR}$ and ${\overline{V(t)}}_{SP}$ is shown as a function of cardiac cycle.

Flow probes and PC-CMR were used to acquire flow measurements into and out of the LV. The volume of the ventricle as a function of time was calculated from each of these modalities and was compared to the values obtained from SP and CMR respectively (Fig. 9). It was found that the average discrepancy (across the cardiac cycle) between SP and the flow probes was 5.9 ± 4.1 % while that between CMR and PC-CMR was found to be 8 ± 6 %. Figure 10 compares the normalized volume of the LV calculated from CMR and SP. The qualitative trends through the cardiac cycle were similar. The mean discrepancy between CMR and SP was 5.5 ± 3.7 % while the largest discrepancy throughout the entire cardiac cycle was approximately 14 %, occurring during peak diastole and peak systole.

## Discussion

The results of this study demonstrated the applicability of an CMR-compatible LV physical model, for the dual objectives of: (a) developing a test bed to validate volume reconstruction methods used to process clinical CMR data, via comparison with higher resolution data acquired using modalities available at the laboratory level, and (b) acquiring ventricular wall motion using CMR sequences for use as initial conditions in LV FSI models, in order to validate the predictive accuracy of the simulations through comparison with CMR-based flow field data.

Though a considerable number of experimental studies have used *in vitro* LV models [12–18, 35], no study to date has used such a platform to provide a one-to-one link between data obtained using laboratory-level experimental modalities (which are of higher fidelity and accuracy) and CMR. Furthermore, no study has examined validation of reconstruction methods used to characterize LV wall motion from CMR data. These two aspects are specifically important when considering *in vitro* platforms for validating LV FSI models that use CMR data as an input for simulations. The study presented in this paper presents the first step in these important directions, with the ultimate objective of translating computational models of the left heart to use in clinical practice and patient-specific planning of therapeutic and surgical interventions.

We have shown that the novel CMR-compatible LV physical model developed in this paper is able to closely simulate the physiological hemodynamic environment of the LV. By adjusting the systemic resistance and compliance elements in the flow loop, we have demonstrated the capability of the LV physical model to match physiological flow rates through the MV and AV, and aortic and ventricular pressures. The intra-ventricular flow field observed within the LV physical model using planar DPIV showed the formation of a counter-rotating vortex ring structure with a trailing jet. This flow pattern qualitatively matches with previous *in vivo* results based on flow fields obtained from CMR [4, 6, 8, 11] and echocardiographic [7, 9, 10] data in healthy volunteers.

The motion of markers embedded on the LV model was tracked using SP and 3D wall motion was reconstructed for the entire cardiac cycle, and this representation was compared to CMR-data based reconstruction of ventricular wall motion. The normalized volume of the LV was used as the metric of comparison between SP, CMR, bulk hemodynamic data obtained from the flow probes and PC-CMR modalities. The comparisons demonstrated good agreement between the multiple modalities (less than 9 % average difference in normalized volume between SP and CMR) in characterizing the wall motion, thus validating the CMR motion reconstruction method employed in this study.

To understand the reasons for mismatch between SP and CMR reconstruction results, it is instructive to consider the different sources of error between SP and CMR motion reconstruction. SP relies on tracking the pixel location of markers and constructing point clouds of the geometry in time. The calibration method used for SP can affect the accuracy of the linear mapping between object-space coordinates and image-plane coordinates. The LDLT method, which discretizes the control volume of the calibration space into smaller volumes [33, 36], was used in this study to minimize nonlinearities in the refracted point coordinates due to imaging through multiple fluid media (air-acrylic-water/glycerin). However, any mismatch between the positions of the SP calibration acrylic tank and the LV chamber can result in calibration errors. We ensured that this mismatch was minimized during SP experiments via placing the calibration tank and LV chamber within identical constraining brackets.

For the case of anatomical CMR measurements, blurring of the LV model wall close to the start of diastole as well as peak systole was observed, mainly due to insufficient temporal resolution. We employed a modified intensity-based edge detection algorithm [34] for segmentation of anatomical images, and these methods typically encounter difficulty in following the tracked boundary accurately due to heterogeneities in intensity values near the edge. As a result, this resulted in ambiguity in identifying the border during segmentation of some of the cardiac phases where blurring was clearly observed. These factors can contribute to the mismatch of CMR-based volume values when compared to SP and flow probe based calculations. The mean error between the values of normalized volume determined using CMR and SP modalities was 5.5 ± 3.7 %, thus demonstrating a reasonable matching considering all the above sources of error. The largest errors between these two modalities were on the order of ~13 %. This was most likely due to the fact that only 20 phases/cycle of PC-CMR data was used to reconstruct the volume into and out of the LV.

In addition to being able to provide experimental data for FSI model verification and validation applications, the CMR-compatible *in vitro* platform allows for conducting controlled studies to compare accuracies of the various post-processing methods used to reconstruct anatomical motion from CMR-data. Such a validation is critical for determining the accuracies of CMR reconstruction methods [28], which in turn are used to provide wall motion input data for computational models [16, 21, 24, 37]. Simultaneously, this model provides CMR scientists with a flexible platform to develop and test various CMR sequences to improve spatiotemporal resolution, while optimizing for the minimal time required for scans.

A number of limitations should be considered while interpreting the results of this study. Though the hemodynamic environment of the simulator was tuned to mimic physiological values, the actual wall motion of the LV model and LV stiffness were not matched to exactly mimic *in vivo* LV wall motion. The anterior and inferior walls of the LV model contracted to a greater extent as compared to the lateral and septal walls. These uneven contractions lead to greater acceleration of the anterior and posterior walls to an extent that CMR protocol used was not able to capture the wall at its highest velocity, resulting in blurred images. These limitations are chiefly attributed to the elastomeric material used for the model design. However, the comparisons between modalities presented here are relative to each other, and therefore will not be impacted by any concerns of imprecise matching to physiological LV wall motion. Future studies will focus on material optimization for obtaining more physiologically realistic wall motion. The biphasic flow of LV filling was not modeled in order to simplify the motion of the LV wall. Similarly, because this was a comparative study, the results reported in this work will not be impacted by this limitation. Only 20 phases/cycle were obtained for the PC-CMR acquisition. This relatively low temporal resolution could be another source of the larger discrepancies when this modality was compared to its counterparts. Finally, this work was not intended for the extensive comparisons between flow fields derived from PC-CMR and DPIV; the comparisons presented here was only used to show that PC-CMR was able to capture the bulk flow structures within the LV.

## Conclusions

In conclusion, the CMR-compatible *in vitro* model developed in this study allows for systematic comparison between laboratory and clinical imaging modalities. It has been used to compare the normalized volume between the CMR and SP modality. This model can also be used as a test-bed for the optimization and validation of new CMR sequences. Additionally, the versatility of the LV model is further enhanced due to its modular design. It is possible to use this model to characterize hemodynamic consequences due to the alteration of anatomical variables (valvular and ventricular: for example, LV wall stiffness, aortic/mitral valve insufficiency or stenosis, etc.). Also, the use of a programmable piston pump to drive ventricular wall motion allows for varying heart rates and heart rate regularities to be set. All these variables enable the LV model provide a physical understanding of structural heart disease and valvular/pathological conditions affecting cardiac function, in order to identify novel diagnostic indices and potential target variables to monitor during treatment.
